# Supplementary material for: Resolving the full spectrum of human genome variation using Linked-Reads
Source: Genome Res. 2019 Apr;29(4):635–45. doi: 10.1101/gr.234443.118 (PMC6442396; doi:10.1101/gr.234443.118)
Supplement: Supplemental Material [file supp_gr.234443.118_Supplemental_longranger-2.2.zip › longranger-2.2.tar.bz2!/lr2.2-public/lib/stan/cmdstan/stan/doc/api/html/stan_8dox.html]

Stan: src/doxygen/stan.dox File Reference


|  |  |
| --- | --- |
|  | Stan  2.5.0  probability, sampling & optimization |


- Main Page
- Related Pages
- Namespaces
- Classes
- Files

- File List
- File Members

All Classes Namespaces Files Functions Variables Typedefs Enumerations Enumerator Friends Macros Pages

Namespaces

src/doxygen/stan.dox File Reference

|  |  |
| --- | --- |
| Namespaces | |
|  | stan |
|  | Probability, optimization and sampling library. |
|  | |
|  | stan::agrad |
|  | Function gradients via reverse-mode automatic differentiation. |
|  | |
|  | stan::gm |
|  | Directed graphical model compiler. |
|  | |
|  | stan::io |
|  | Input/output utilities. |
|  | |
|  | stan::math |
|  | Matrices and templated mathematical functions. |
|  | |
|  | stan::math::policies |
|  | Extending boost functionality. |
|  | |
|  | stan::math::policies::detail |
|  | Reimplementing boost::math::policies::detail::raise\_domain\_error. |
|  | |
|  | stan::mcmc |
|  | Markov chain Monte Carlo samplers. |
|  | |
|  | stan::memory |
|  | (Expert) Allocate, free, and manage underlying system memory. |
|  | |
|  | stan::model |
|  | For compiling models. |
|  | |
|  | stan::prob |
|  | Templated probability distributions. |
|  | |


---

[ Stan Home Page ]

*© 2011–2014,
Stan Development Team.*
